# Nitrogen addition influences tomato resistance to the destructive invasive pest *Tuta absoluta*

**DOI:** 10.1093/hr/uhaf099

**Published:** 2025-03-25

**Authors:** Zichao Song, Cheng Qu, Ziying Liu, Ruiyan Piao, Aoli Lin, Xiaofan Yang, Chuanyou Li, Jianghua Sun, Zhiwei Kang

**Affiliations:** College of Life Science/Hebei Basic Science Center for Biotic Interactions, Institute of Life Science and Green Development, Hebei University, No. 180 Wusi Dong Road, Baoding 071002, China; Beijing Key Laboratory of Environment Friendly Management on Fruit Diseases and Pests in North China, Institute of Plant Protection, Beijing Academy of Agriculture and Forestry Sciences, No. 9 dawn Garden Road, Beijing 100081, China; College of Life Science/Hebei Basic Science Center for Biotic Interactions, Institute of Life Science and Green Development, Hebei University, No. 180 Wusi Dong Road, Baoding 071002, China; College of Life Science/Hebei Basic Science Center for Biotic Interactions, Institute of Life Science and Green Development, Hebei University, No. 180 Wusi Dong Road, Baoding 071002, China; College of Life Science/Hebei Basic Science Center for Biotic Interactions, Institute of Life Science and Green Development, Hebei University, No. 180 Wusi Dong Road, Baoding 071002, China; Plant Protection Institute, Hebei Academy of Agriculture and Forestry Sciences, No. 437 Dongguan Road, Baoding 071000, Hebei, China; College of Life Sciences, Shandong Agricultural University, No. 61 Daizong Street, Tai’an 271018, Shandong, China; College of Life Science/Hebei Basic Science Center for Biotic Interactions, Institute of Life Science and Green Development, Hebei University, No. 180 Wusi Dong Road, Baoding 071002, China; College of Life Science/Hebei Basic Science Center for Biotic Interactions, Institute of Life Science and Green Development, Hebei University, No. 180 Wusi Dong Road, Baoding 071002, China; College of Life Sciences, Shandong Agricultural University, No. 61 Daizong Street, Tai’an 271018, Shandong, China

Dear Editor,

As one of the most widely planted and popular vegetables, tomatoes are highly susceptible to a range of insect pests and diseases that significantly affect their growth, yield, and fruit quality. Nitrogen (N), a critical nutrient for plant growth and development, plays a complex role in plant defense mechanisms against pathogens and pests, with its effects appearing highly variable [1, 2]. The South American tomato pinworm, *Tuta absoluta* Meyrick is one of the most destructive invasive pests causing serious economic losses on tomato production globally. It originated from South America but then rapidly and aggressively invaded more than 110 countries over Europe, Africa, and Asia (EPPO, https://gd.eppo.int/taxon/GNORAB/distribution). Now, it has been considered as the major threat to global tomato production. Previous study has shown that nitrogen availability to tomato plants affected the juvenile survival rate and development of *T. absoluta*, but the molecular mechanisms underlying these effects remain poorly understood [3].

In this study, we found high N (HN; 10 mM urea) input significantly increased the larval feeding activity of *T. absoluta* (Fig. 1A). Consistent with the enhanced larval feeding, the pupal weight of *T. absoluta* reared on HN-treated tomato plants was significantly heavier than that on normal nitrogen (NN; 20 mM)-treated plants, which is consistent with the previous study [3] (Fig. 1B). Interestingly, HN treatment significantly inhibited the oviposition preference of *T. absoluta* adult females (Fig. 1C), a finding that contrasts with observations in the brown planthopper, *Nilaparvata lugens* [2]. Specifically, Sun *et al.* [2] reported that brown planthoppers preferred to lay eggs on HN-treated rice plants. Taken together, these results suggest that HN treatment enhances larval performance but reduces oviposition preference in female *T. absoluta*.

**Figure 1:**
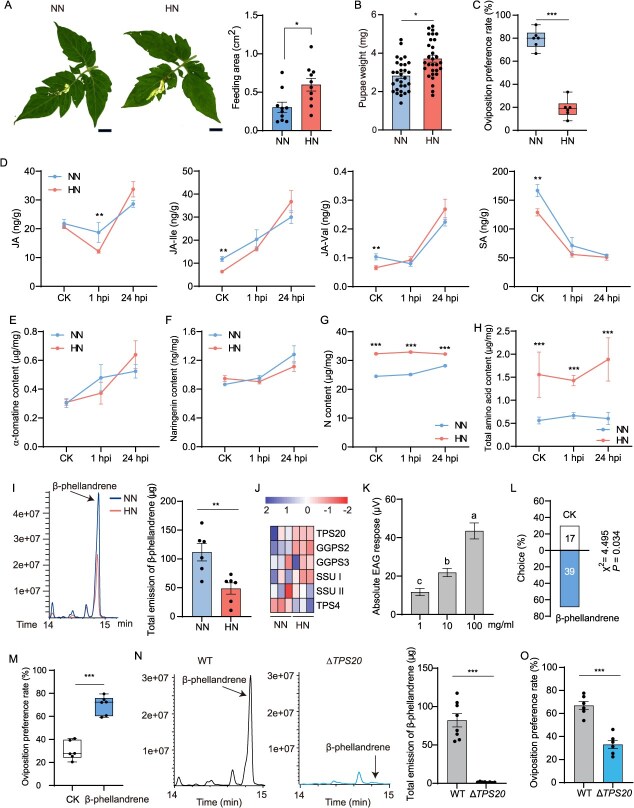
Nitrogen addition benefits the larval performance of *T. absoluta* but inhibits its oviposition preference in adult stage. (A) Feeding area of *T. absoluta* larvae on HN- and NN-treated tomato plants. Scale bar = 1 cm. (B) Pupae weight of *T. absoluta* reared on HN- and NN-treated tomato plants. (C) Adult female oviposition preference of *T. absoluta* between HN- and NN-treated tomato plants. (D) Phytohormone levels in HN- and NN-treated tomato plants (*n* = 5). Effect of HN and NN treatment on the accumulation of α-tomatine (E) and naringenin (F) in tomato leaves. Leaf N (G) and total amino acids (H) content in HN- and NN-treated tomato plants. (I) Total emission of β-phellandrene in HN- and NN-treated tomato plants. (J) Expression of genes involved in β-phellandrene biosynthesis in HN- and NN-treated tomato plants. (K) The EAG response of female antennae to β-phellandrene. (L) Behavioral responses of *T. absoluta* female adults to β-phellandrene (*n* = 60 individuals). (M) Effect of β-phellandrene supplementation on the oviposition preference of *T. absoluta* female adults. (N) Effect of *TPS20* deletion on the emission of β-phellandrene. (O) *TPS20* deletion decreased the adult female oviposition preference of *T. absoluta*.

To investigate why HN input benefits the larval growth of *T. absoluta*, we firstly analyzed the levels of plant defense hormones (jasmonic acid, JA and salicylic acid, SA) in tomato plants with HN or NN input following *T. absoluta* infestation. The results showed that HN input significantly suppressed the levels of two JA amino acid conjugates, jasmonoyl-L-isoleucine (JA-Ile), and JA-valine (JA-Val), as well as SA in healthy tomato plants (Fig. 1D). Infestation by *T. absoluta* larvae significantly decreased the JA content in HN-treated tomato plants at 1 hour postinfestation (hpi). However, by 24 hpi, there was no significant differences in JA levels between HN- and NN-treated plants (Fig. 1D). At 24 hpi, *T. absoluta* larvae infestation significantly induced the accumulation of JA and its amino acid conjugates in both HN- and NN-treated plants (Fig. 1D). In contrast, SA content was significantly reduced by *T. absoluta* larvae infestation at both 1 and 24 hpi (Fig. 1D).

In alignment with the JA content, infestation by *T. absoluta* larvae significantly induced the accumulation of α-tomatine (Fig. 1E) and naringenin (Fig. 1F) in tomato leaves, which are two known plant defensive compounds. In contrast, nitrogen input showed no significant influence on this accumulation. Nitrogen is a foundational element for amino acid synthesis, serving as a precursor for glutamate and glutamine-central hubs in plant N metabolism. HN treatment notably increased the N (Fig. 1G) and total amino acids (Fig. 1H) content in tomato leaves. This nutrient enrichment creates a high-quality food source for herbivores, as amino acids are essential for larval development, particularly for synthesizing proteins and enzymes required for growth and metamorphosis. Collectively, these findings indicate that HN suppresses some defense hormones (JA conjugates and SA) under healthy conditions, but upon infestation, the defenses (JA, conjugates, α-tomatine, naringenin) are induced similarly in both HN and NN. However, HN increases nutrients (N and amino acids), which might benefit the larvae despite the defenses.

To investigate the impact of HN input on the oviposition preference of *T. absoluta* female adults, we analyzed the differences in volatiles emissions between HN- and NN-treated tomato plants. The results revealed that the emission of β-phellandrene, the most abundant volatile compound released by tomato plants, was significantly lower in HN-treated tomato plants compared to NN-treated plants (Fig. 1I). In line with the volatile emission data, transcriptome sequencing of HN- and NN-treated tomato plants also demonstrated significantly reduced expression levels of genes associated with β-phellandrene biosynthesis in HN-treated tomato plants (Fig. 1J). Based on these findings, we hypothesize that HN-treatment significantly inhibits the oviposition preference of *T. absoluta* females by suppressing the emission of β-phellandrene.

To determine whether β-phellandrene plays a role in the oviposition preference of *T. absoluta* female adults, we firstly examined the electroantennograms (EAG) response of *T. absoluta* females to β-phellandrene. The results showed β-phellandrene elicited a significant antennal response in *T. absoluta* females (Fig. 1K). Y-tube experiments using synthetic β-phellandrene confirmed its attractiveness to *T. absoulta* females (Fig. 1L). Additionally, supplementation of β-phellandrene significantly increased the oviposition preference of *T. absoluta* females (Fig. 1M). To further investigate the role of β-phellandrene, we generated a mutant of terpene synthase 20 (*TPS20*), a key gene in β-phellandrene biosynthesis [4], using CRISPR-cas9. Volatile emission analysis revealed that Δ*TPS20* plants released significantly lower amount of β-phellandrene compared to WT plants (Fig. 1N). Importantly, the deletion of *TPS20* significantly reduced the oviposition preference of *T. absoluta* females (Fig. 1O). Collectively, these findings demonstrate that β-phellandrene is critical plant volatile for host location and oviposition in *T. absoluta* females. Furthermore, recent studies have highlighted the potential of manipulating plant volatiles through gene-editing and transgenic approaches as a new and environmentally friendly strategy for pest management [5]. For example, overexpression of *CsCYP82L2* to produce (3E)-4,8-dimethyl-1,3,7-nonatriene and (E,E)-4,8,12-trimethyl-1,3,7,11-tridecatetraene significantly reduced the Asian citrus psyllid (*Diaphorina citri* Kuwayama) preference [5]. Therefore, our results suggest that *TPS20* represents a promising target for the control of *T. absoluta* through the modulation of β-phellandrene emission.

In summary, our study demonstrates that HN treatment not only enhances the growth of tomato plants but also benefits the larval development of *T. absoluta*. However, HN treatment was found to inhibit the oviposition preference of *T. absoluta* females. Analysis of plant defense revealed that HN input reduced the levels of plant defense hormones but did not affect the defense induction triggered by *T. absoluta* infestation. Conversely, changes in plant nutritional quality indicated higher N and amino acid content in HN-treated tomato plants. These finding suggest that improved nutritional quality, rather than plant defense, is the primary factor driving better larval performance on HN-treated plants. Furthermore, we established that the reduced oviposition preference of *T. absoluta* females on HN-treated plants is associated with the lower emission of β-phellandrene in these plants. Collectively, our results elucidate the impact of HN input on the interaction between tomato plants and *T. absoluta*, providing mechanistic insights into how nitrogen influences insect–plant interactions. The findings from this study offer valuable information for optimizing N application in agriculture and highlight the important of finding an appropriate nitrogen application strategy that not only promotes plant growth but also improves plant resistance to plant pests. Moreover, long-term field experiments about nitrogen fertilization on plant growth and defense are also required. Our results also provide a new management strategy of *T. absoluta* through the modulation of plant volatile emissions.

## Data Availability

Transcriptome data has been uploaded into the SRA database in NCBI (BioProject ID: PRJNA1222811).
